# S100A9: A Potential Biomarker for the Progression of Non-Alcoholic Fatty Liver Disease and the Diagnosis of Non-Alcoholic Steatohepatitis

**DOI:** 10.1371/journal.pone.0127352

**Published:** 2015-05-19

**Authors:** Xiaolin Liu, Yongfeng Wang, Yanan Ming, Yanyan Song, Jingyi Zhang, Xiaoyu Chen, Minde Zeng, Yimin Mao

**Affiliations:** 1 Division of Gastroenterology and Hepatology, Renji Hospital, School of Medicine, Shanghai Jiao Tong University, Shanghai Institute of Digestive Disease, Shanghai, China; 2 Department Biostatistics, Institute of Medical Sciences, Shanghai Jiao Tong University School of Medicine, Shanghai, China; INRA, FRANCE

## Abstract

Non-alcoholic fatty liver (NAFL) has the potential to progress to non-alcoholic steatohepatitis (NASH) or to promote type 2 diabetes mellitus (T2DM). However, NASH and T2DM do not always develop coordinately. Additionally, there are no definite noninvasive methods for NASH diagnosis currently. We established rat models of NAFL, NASH, and NAFL + T2DM to recapitulate different phenotypes associated with non-alcoholic fatty liver disease (NAFLD) and its progression. Histologic features of rat livers were scored according to criteria established by the Nonalcoholic Steatohepatitis Clinical Research Network. Microarray was performed to assess gene expression changes in rat livers. We find that gene expression of s100a9 was higher in NAFL group compared with control, and was increased in NASH groups and decreased in NAFL + T2DM group compared with NAFL. In contrast, srebf1, tbx21, and gimap4 only showed limited discriminating abilities in different groups. There is a significant positive correlation between serum levels of S100A9 and NAFLD Activity Score (NAS), the severity of hepatic steatosis, and lobular inflammation (r = 0.80, 0.64 and 0.86, *P* < 0.001). These findings suggest that S100A9 may be extremely useful in the diagnosis of NASH (AUROC: 0.947, CI: 0.845-1.049). Additionally, serum S100A9 levels displayed a strong correlation with ALT, AST and TBil (r = 0.81, 0.89 and 0.91, *P* < 0.001) but a weak correlation with FBG, HOMA-IR, TG, and TC (r = -0.41, -0.40, 0.47 and 0.49, *P* < 0.05). Conclusions: The results we provide here suggest that S100A9 may be useful as a biomarker for the hepatic and metabolic progression of NAFLD and the non-invasive diagnosis of NASH.

## Introduction

Non-alcoholic fatty liver disease (NAFLD) has become a major cause of chronic liver disease. It is considered a growing public health concern worldwide, with a prevalence of more than 30% in the general population [1]. Histologically, NAFLD includes both NAFL and non-alcoholic steatohepatitis (NASH) [2]. Though most hepatic steatosis in NAFL is self-limiting, it can progress to NASH, cirrhosis, or even hepatocellular carcinoma [3]. Importantly, a growing body of evidence confirms that NAFLD is associated not only with liver-related morbidity and mortality, but also with an increased risk of developing metabolic syndrome (MS), particularly obesity and diabetes [4, 5]. The major hepatic and extra-hepatic manifestations of NAFLD do not typically coordinately develop in clinical practice. For example, it is not uncommon to find non-obese NAFLD patients [6], and some NAFL patients will progress to NASH without exhibiting features of MS, particularly in Asia [7, 8]. On the contrary, some NAFLD patients suffer from severe obesity and insulin resistance but show little hepatic progression. Although great progress has been made in our understanding of the pathophysiology of NAFLD [3, 9, 10], there is still lack of knowledge concerning the differentiation of advanced NAFLD in its hepatic and metabolic profiles.

There is also great interest in the development and validation of a noninvasive diagnostic method for NASH. Liver biopsy remains the gold standard for disease diagnosis, but its invasive nature and high cost limits its widespread use in clinical practice [11]. Therefore, simpler, more accurate, less invasive, and more affordable screening tools for NASH would be extremely useful. Recent work has described the use of non-invasive biomarkers associated with hepatocyte apoptosis (cytokeratin-18, CK-18), inflammation (ferritin), and fibrosis (procollagen III N-terminal propeptide, PIIINP), and other types of scoring systems and imaging methods to diagnose NASH [12, 13]. However, these approaches are not sensitive or specific enough to act as robust predictors of NAFLD in isolation. Importantly, high throughput methods, such as transcriptomics and proteomics, facilitate the identification of more specific, mechanism-based biomarkers to distinguish different phenotypes of NAFLD.

In the present study, we developed NAFL [14], NASH [15], and NAFL + T2DM [16, 17], rat models to mimic human simple liver steatosis, steatohepatitis without MS, and hepatic steatosis accompanied with T2DM, respectively. Using microarrays, we have identified hepatic gene expression changes in each of these models. The goal is to identify a predictor of different NAFLD progressions, as well as provide a new diagnostic marker for NASH.

## Materials and Methods

### Experimental animals and sample collection

**Fig 1:**
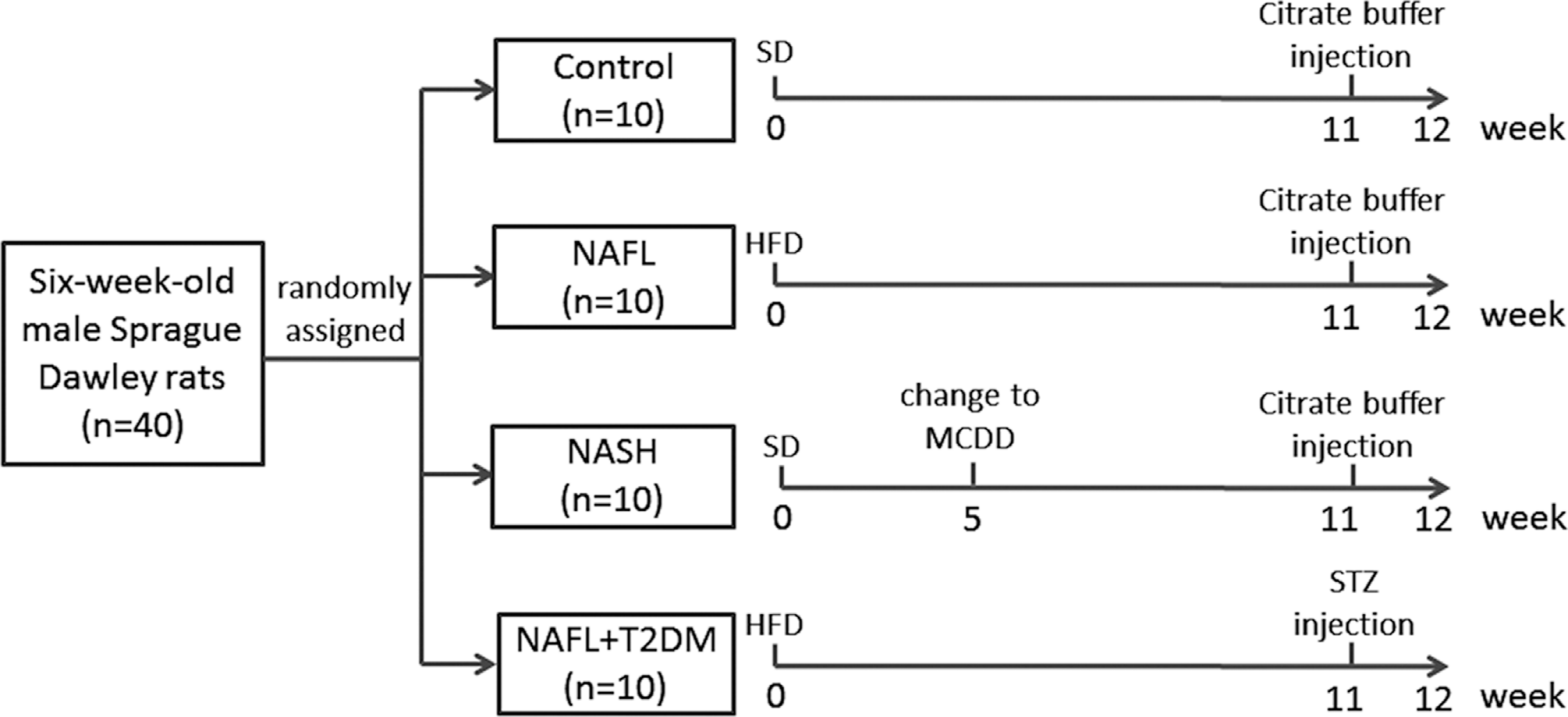
The flow chart of modeling control, NAFL, NASH and NAFL + T2DM rats. The STZ (25 mg/kg in 0.1 M citrate buffer) and citrate buffer were given to rats through a single intraperitoneal injection. SD, standard diet; HFD, high fat diet; MCDD, methionine-choline-deficient diet; STZ, streptozotocin.

Six-week-old male Sprague—Dawley rats were purchased from the Shanghai Experimental Animal Center of the Chinese Academy of Sciences (Shanghai, China) and maintained under controlled conditions of temperature (24° ± 2°C), humidity (50% ± 5%), and 12 hour light-dark cycles. Animals had free access to food and water throughout the experimental period. After acclimation for one week on a standard diet (SD), rats were randomized into four experimental groups (10 rats/group) and fed for 12 weeks as follows: (1) control: standard diet (SD); (2) NAFL: high fat diet (HFD; D12492, Research Diets, USA); (3) NASH: received SD for the first four weeks and then switched to a methionine-choline-deficient diet (MCDD; A02082002B, Research Diets, USA) until the end of the experiment; and (4) NAFL + T2DM: HFD (D12492, Research Diets, USA) and a single intraperitoneal injection of streptozotocin (STZ; Sigma, USA; 25 mg/kg in 0.1 M citrate buffer) at the end of week 11. Rats in the other groups received a single intraperitoneal injection of 0.1 M citrate buffer (25 mg/kg) as a control (Fig 1). Liver biopsies indicating simple hepatic steatosis with a NAFLD Activity Score (NAS) < 5 were diagnosed as NAFL; NASH was defined as significant hepatic steatosis with hepatocellular injury and NAS ≥ 5 [2, 18]. Fasting blood glucose (FBG) levels ≥ 11.1 mmol/L in two consecutive measurements following STZ injection indicated successful establishment of the T2DM model [19].

Body weights of rats were recorded weekly. Three days after STZ administration, FBG was measured using a Roche Blood Glucometer (ACCU-CHEK Active, Roche, Germany). At the end of week 12, rats were euthanized after a period of overnight fasting. Blood was drawn from the retinal vein plexus and then centrifuged; serum was frozen at -80°C for subsequent measurement. Whole liver was removed and weighed to calculate the liver index (liver weight/body weight). A small part of the liver was fixed in 4% formaldehyde overnight and embedded in paraffin wax for histological assessment. The remaining portion was frozen in liquid nitrogen and stored at -80°C for later analysis.

All animal experiments were approved by the Institutional Animal Care and Use Committee of Shanghai Jiao Tong University School of Medicine and were conducted in accordance with the National Research Council Guide for Care and Use of Laboratory Animals.

### Histological examination

Paraffin sections of livers were stained with hematoxylin-eosin (H&E), and frozen sections of livers were stained with oil red O. Pathological features of steatosis (0–3), lobular inflammation (0–2), hepatocellular ballooning (0–2), and fibrosis (0–4) were scored by an experienced pathologist, according to criteria established by the Nonalcoholic Steatohepatitis Clinical Research Network [18]. NAS was calculated by adding the scores of steatosis, lobular inflammation, and hepatocellular ballooning.

### Biochemical assays

Serum alanine aminotransferase (ALT), aspartate aminotransferase (AST), total bilirubin (TBil), triglyceride (TG), total cholesterol (TC), high-density lipoprotein (HDL), low-density lipoprotein (LDL), and FBG were measured using an automatic biochemical analyzer (SIEMENS ADVIA 1800: SIEMENS Healthcare Diagnostics, USA). Fasting insulin (FINS) was measured using a gamma radioimmunoassay counter (SN-697, Hesuo Rihuan, China). The homeostasis model assessment of insulin resistance (HOMA-IR) was calculated as FBG x FINS/22.5.

### Microarray gene expression studies

The liver samples used for microarray analysis were obtained from 12 rats (3 samples being randomly selected from each group). Total RNA was isolated from rat livers using the standard Trizol method (Takara, Shiga, Japan). Gene expression experiments were performed using microarrays containing 24,358 genes, according to the manufacturer’s instructions (Phalanx Rat One Array Plus Genome). Raw intensity signals of each microarray were scanned with GenePix Personal 4000B (Molecular Devices Corporation, Sunnyvale, CA, USA) and quantified using GenePix Pro 4.0 software (Molecular Devices Corporation, Downingtown, PA, USA). Genes showing more than a 2-fold change between two groups were selected for further analysis. Gene Set Enrichment Analysis (GSEA) was used to perform pathway and gene ontology analysis. The gene expression information in this experiment had been submitted to the Gene Expression Omnibus database with the registration No. GSE65220.

### Quantitative real-time PCR

Quantitative real-time polymerase chain reaction (qRT-PCR) was performed on select genes to verify the microarray analysis results. RNA was extracted from all rat livers as described above. Complementary DNA was synthesized using PrimeScript RT Reagent Kit (Takara, Shiga, Japan), according to the manufacturer’s instructions. SYBR Premix Ex Taq II (Takara, Shiga, Japan) was used for qRT-PCR. The mRNA abundance of tested genes was normalized to that of 18s and expressed relative to control. Sequences of primers used were: srebf1: 5’-GGATGCTCGTGCCAGTG-3’, 5’-ACTCAGTGCCAGGTTAGAAG-3’; tbx21: 5’-AGCCGTTTCTACCCTGACCT-3’, 5’-CTGCTCGGAACTCTGTTTCA-3’; gimap4: 5’-GGCTTTGCCTTCTAATGGTG-3’, 5’-GGTTCCTGCTTTGAGTTGCT-3’; s100a9: 5’-GAGGAGTGTATGATGCTGATGG-3’, 5’-GACTTGGTTGGGCAGATGTT-3’; 18s: 5’-AAGTTTCAGCACATCCTGCGAGTA-3’, 5’-TTGGTGAGGTCAATGTCTGCTTTC-3’.

### Western blotting

Total protein from rat livers was extracted using RIPA lysis buffer (Beyotime, China) at 4°C. The protein concentration was measured using a Pierce BCA Protein *Assay Kit (Thermo Scientific, USA). Protein samples of 50 μg each were separated on 12% SDS-polyacrylamide gel and electro-transferred to nitrocellulose membranes. The membranes were blocked with 5% skim milk in TBST for 1 h at room temperature, then incubated with mouse monoclonal antibody against rat SREBF1 (Abcam, Cambridge, UK; diluted 1:500 in TBST); rabbit polyclonal antibody against rat TBX21 (CUSABIO, Wuhan, China; diluted 1:200 in TBST); goat polyclonal antibody against rat CIMAP4 (Santa Cruz, Texas, USA; diluted 1:200 in TBST); rabbit polyclonal antibody against rat S100A9 (Abcam, Cambridge, UK; diluted 1:1000 in TBST) overnight at 4°C. β-actin was used as the internal control. After a subsequent washing step in TBST, the membranes were incubated with HRP-conjugated secondary antibody (Beyotime, China) at room temperature for 2 h. The signals were detected with a Pierce ECL Western Blotting Kit (Thermo Scientific, USA).

### Measurement of serum SREBF1, TBX21, GIMAP4, S100A9 and CK18 Levels by ELISA

Serum levels of some proteins in rats were measured using the Rat SREBF-1 Elisa kit, Rat tbx-21 Elisa kit, Rat gimap-4 Elisa kit (Blue Gene, Shanghai, China), Rat Protein S100A9 ELISA kit and Rat CK-18 ELISA kit (CUSABIO, Wuhan, China). 100 μL of each serum sample was placed in each well. The wells were sealed with an adhesive strip, and the plate was then incubated for 2 h at 37°C. Next, 100 μL of biotin-antibody was added to each well and incubated for 1 h at 37°C. After washing in wash buffer 3 times, 100 μL of HRP-avidin was added to each well, which was further incubated for 1 h at 37°C. After washing 5 times, each well was filled with 90 μL of TMB substrate and then incubated for 20 min at 37°C. Finally, 50 μL of stop solution was added to each well. The optical density at a wavelength of 450 nm was determined using a spectrophotometer (Thermo Fisher Scientific, USA). Serum S100A9 concentrations were calculated based on the standard curve.

### Statistical analysis

The data from all experiments, with the exception of the microarray data, are presented as the mean ± SEM. Statistical comparison was made between groups using one-way ANOVA followed by a Newman—Keuls post test. Linear regression analysis was employed to identify the correlation between serum S100A9 and selected parameters. *P* < 0.05 was considered to be statistically significant. Calculations were performed using SPSS version 16.0 (SPSS, Chicago, IL, USA) and GraphPad Prism Software version 5.0 (GraphPad Software, San Diego, CA, USA).

## Results

### Establishing rat models of NAFL, NASH, and NAFL + T2DM

**Fig 2:**
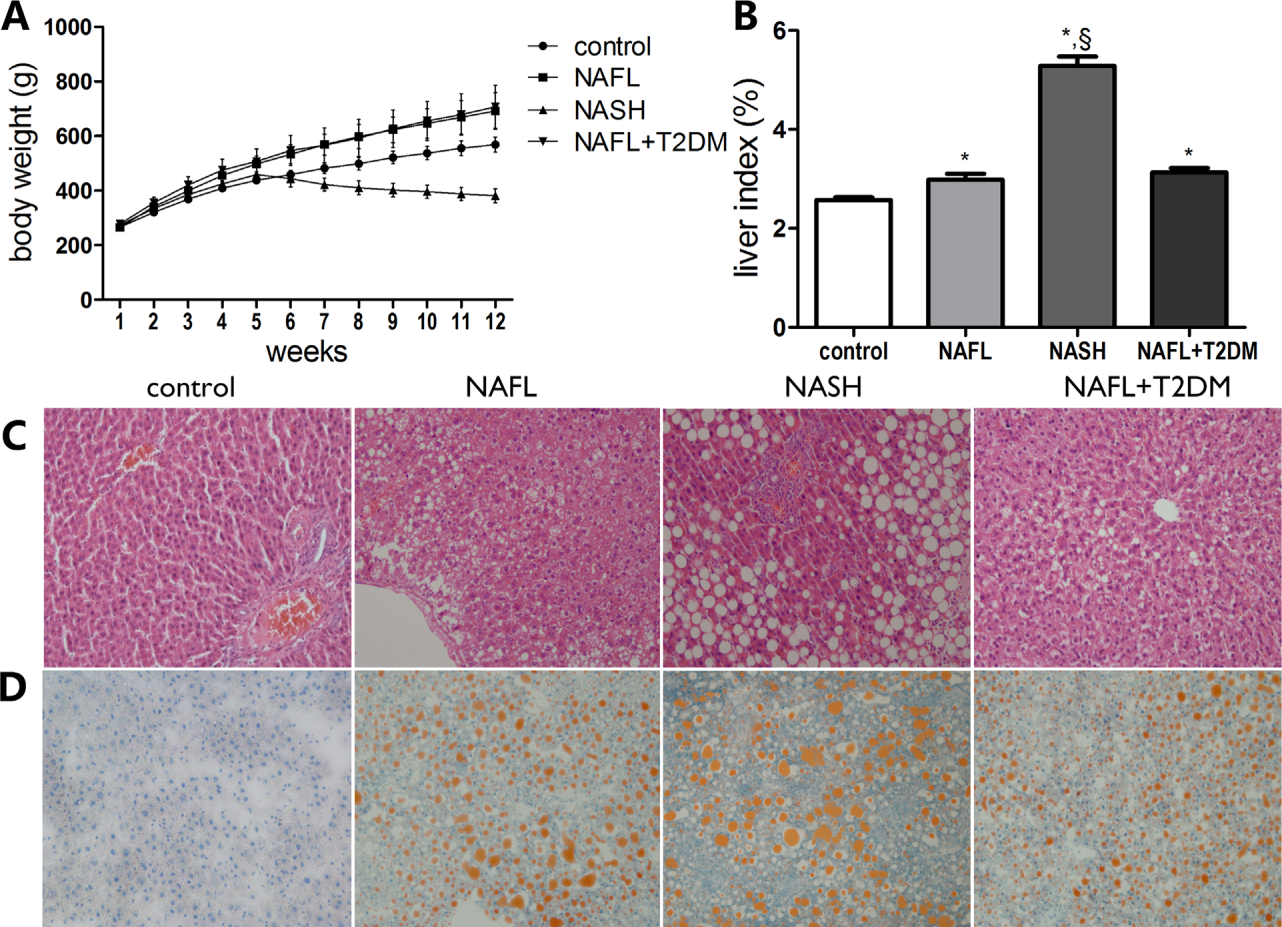
The establishment of rat models in NAFL, NASH and NAFL + T2DM. (A) Changes in body weight of rats during standard diet (control), high fat diet (NAFL and NAFL + T2DM) and methionine-choline-deficient diet (NASH) feeding. (B) Liver index (the ratio of liver weight to body weight) of the rats at the end of the experiment. Liver histological examination with H&E (x 200) (C) and Oil-Red-O staining (x 200) (D). Data are expressed as the mean ± SD (*n* = 10 per group). *P < 0.05 compared with the control group; §*P* < 0.05 compared with the NAFL group.

Rat body weights were measured at the end of the experiment. Rats in both the NAFL and NAFL + T2DM groups had gained significantly more weight by the end of the experiment compared with rats in the control group (*P* < 0.05). In contrast, rats in the NASH group displayed decreased body weight after the MCDD (*P* < 0.05; Fig 2A). The liver index, which served as an indicator of liver injury, increased significantly in all experimental groups; specifically, there was a 2-fold increase in the NASH group compared with the control (*P* < 0.05; Fig 2B).

H&E and oil red O staining showed diffusely mixed hepatic steatosis in rats in both the NAFL and NAFL + T2DM groups; it was primarily zone 3 hepatocytes that were affected. Rats in the NASH group exhibited more significant steatosis, which was accompanied by hepatocellular ballooning, lobular inflammation, and mild perisinusoidal fibrosis (Fig 2C and 2D). The range of NAS in the control, NAFL, NASH, and NAFL + T2DM groups was 0–1, 2–4, 5–7, and 3–5, respectively (S1 Table). Serum levels of ALT (10-fold increase) and AST (5-fold increase) were significantly increased in the NASH group compared with both the control and NAFL groups (*P* < 0.05). These increases are indicative of severe liver injury in the NASH group. FBG levels and HOMA-IR, used as indicators of metabolic status, were increased in the NAFL and NAFL + T2DM groups compared with the control group (*P* < 0.05), and two FBG measurements, 3 days and 1 week after STZ injection, yielded an FBG > 11.1 mmol/L in each of the NAFL + T2DM rats (Table 1).

**Table 1:** General and biochemical parameters of rats in different groups.

Parameters	Control	NAFL	NASH	NAFL + T2DM
**General parameters**				
Body weight(g)	545.8 ± 12.86	681.0 ± 2 8.47*	313.7± 9.22*,§	657.6 ± 23.81*
Liver weight(g)	14.01 ± 0.35	20.26 ± 1.14*	16.52 ± 0.65§	20.67 ± 1.2*
Liver index(%)	2.57 ± 0.06	2.98 ± 0.12*	5.28 ± 0.19*,§	3.13 ± 0.09*
**Biochemical parameters**				
ALT(U/L)	42.3 ± 2.53	147.9 ± 32.75	422.9 ± 63.58*,§	102.6 ± 16.73
AST(U/L)	107.7 ± 3.5	190.3 ± 22.48	587.9 ± 66.0*,§	171.2 ± 19.2
TBil(umol/L)	2.18 ± 0.17	2.25 ± 0.14	6.24 ± 0.44*,§	1.89 ± 0.11
TC(mmol/L)	1.74 ± 0.16	1.92 ± 0.07	2.09 ± 0.27	1.46 ± 0.1
TG(mmol/L)	0.80 ± 0.06	1.35 ± 0.20*	1.70 ± 0.24*	1.66 ± 0.17*
HDL(mmol/L)	0.37 ± 0.02	0.4 ± 0.01	0.35 ± 0.05	0.37 ± 0.03
LDL(mmol/L)	0.13 ± 0.01	0.38 ± 0.2	0.78 ± 0.17*,§	0.08 ± 0.01
FBG(mmol/L)	4.90 ± 0.12	6.68 ± 0.27*	4.37 ± 0.17	13.65 ± 0.99*,§
FINS(mIU/L)	8.50 ± 0.8	23.73 ± 1.99*	7.06 ± 0.62§	17.66 ± 1.62*,§
HOMA-IR	1.85 ± 0.18	7.15 ± 0.8*	1.35 ± 0.11§	11.29 ± 1.43*,§

Data are expressed as the mean ± SEM. ALT, alanine aminotransferase; AST, aspartate aminotransferase; TBil, total bilirubin; TC, total cholesterol; TG, triglyceride; HDL, high-density lipoprotein; LDL, low-density lipoprotein; FBG, fasting blood glucose; FINS, fasting insulin; HOMA-IR, homeostasis model assessment of insulin resistance.

*P < 0.05 compared with control group

^§^ *P* < 0.05 compared with NAFL group.

### Hepatic global gene expression and pathway analysis

We performed global gene expression analysis from the livers of rats belonging to each of the treatment groups to potentially identify biomarkers of each condition and to obtain new insight into the molecular mechanisms associated with NAFLD progression. The heat map that was produced by unsupervised hierarchical clustering of significantly changed genes revealed that samples clustered well within groups (S1 Fig). In the NAFL group, 219 genes were upregulated and 164 genes were downregulated at least 2-fold (*P* < 0.05). Using the same fold-change and P-value cut-offs, we identified 1968 upregulated genes and 946 downregulated genes in the NASH group, and 226 upregulated genes and 234 downregulated genes in the NAFL + T2DM group.

To identify genes capable of distinguishing hepatic progression from metabolic progression in NAFLD, we focused on genes showing opposite regulation in NASH and NAFL + T2DM compared with NAFL. Sterol regulatory element binding transcription factor 1 (srebf1) was the only gene that increased in NAFL + T2DM but decreased in NASH. S100 calcium binding protein A9 (s100a9), t-box 21 (tbx21), and GTPase IMAP family member 4 (gimap4) were up-regulated in NASH but down-regulated in NAFL + T2DM. Interestingly, among these 4 differentially regulated genes, s100a9 was the only one that also showed a significant difference between the NAFL and control group. Raw data from the microarray analysis showed that the mRNA level of s100a9 had increased 2.5-fold in the NAFL compared with the control group. There was a 3.2-fold increase in NASH, and a 0.4-fold decrease in NAFL + T2DM compared with the NAFL group (*P* < 0.05). These results indicate that s100a9 is a potential biomarker with significant sensitivity in the pathogenesis and prognosis of NAFLD.

### Hepatic expression of s100a9, srebf1, tbx21, and gimap4 determined by real-time qRT-PCR

**Fig 3:**
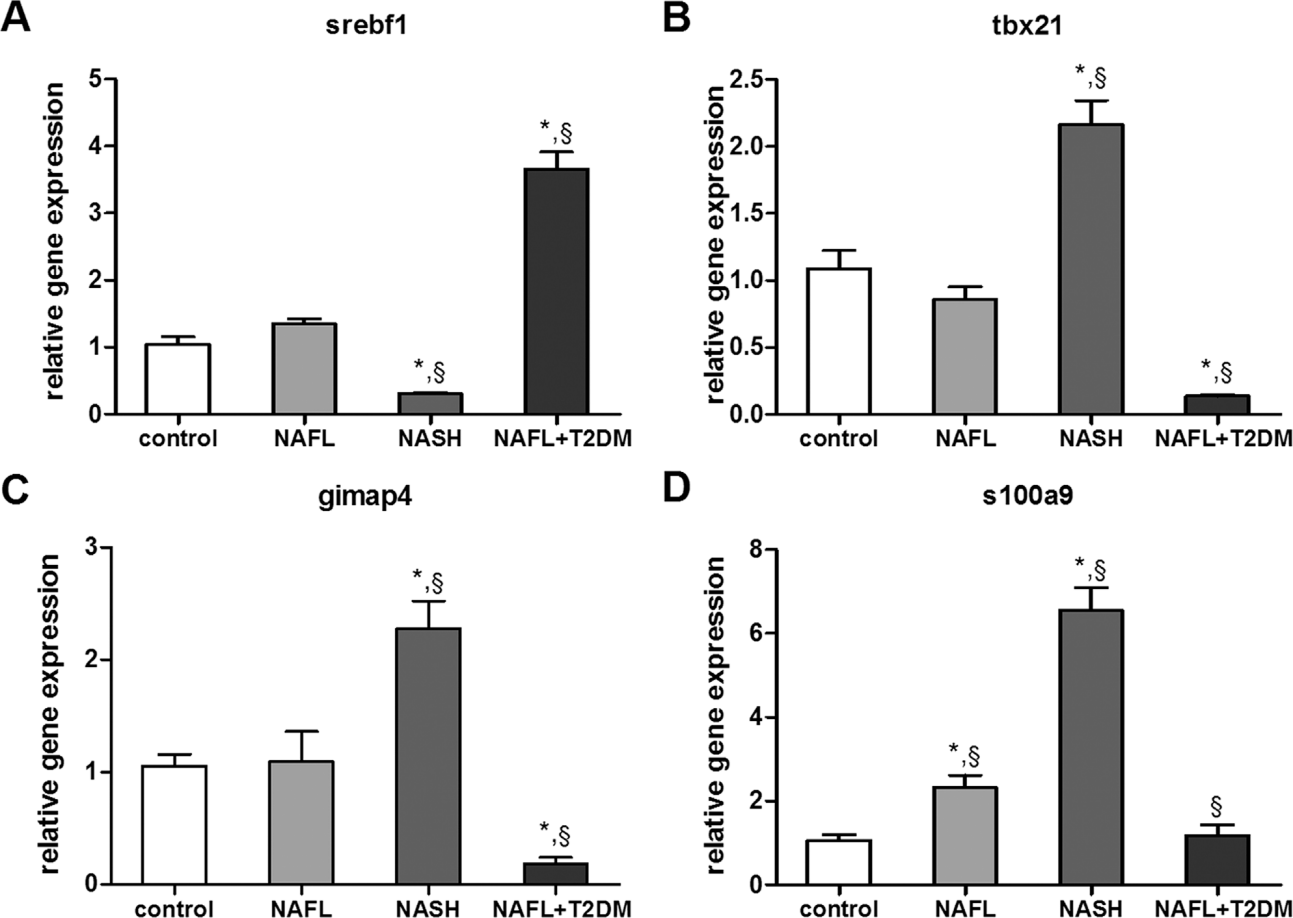
Validation of selected genes in microarray data by qrt-PCR. Hepatic mRNA levels of srebf1 (A), tbx21 (B), gimap4 (C), and s100a9 (D). The mRNA levels were normalized to those of 18S, and subsequently normalized to those of control group. *, *P* < 0.05 compared with the control group; §, *P* < 0.05 compared with the NAFL group.

To validate the results of the microarray experiments, mRNA levels of selected genes were measured by real-time qRT-PCR. Consistent with the microarray data, srebf1, tbx21, and gimap4 showed differential regulation in the NASH and NAFL+T2DM groups compared with the NAFL group. S100a9 increased slightly in the NAFL group compared with the control group (2-fold; *P* < 0.05); when compared with the NAFL group, levels in the NASH groups were significantly higher (3-fold; *P* < 0.05), and levels in the NAFL + T2DM group were slightly decreased (0.5-fold; *P* < 0.05) (Fig 3).

### Hepatic and serum protein levels of SREBF1, TBX21, GIMAP4 and S100A9 in rats

**Fig 4:**
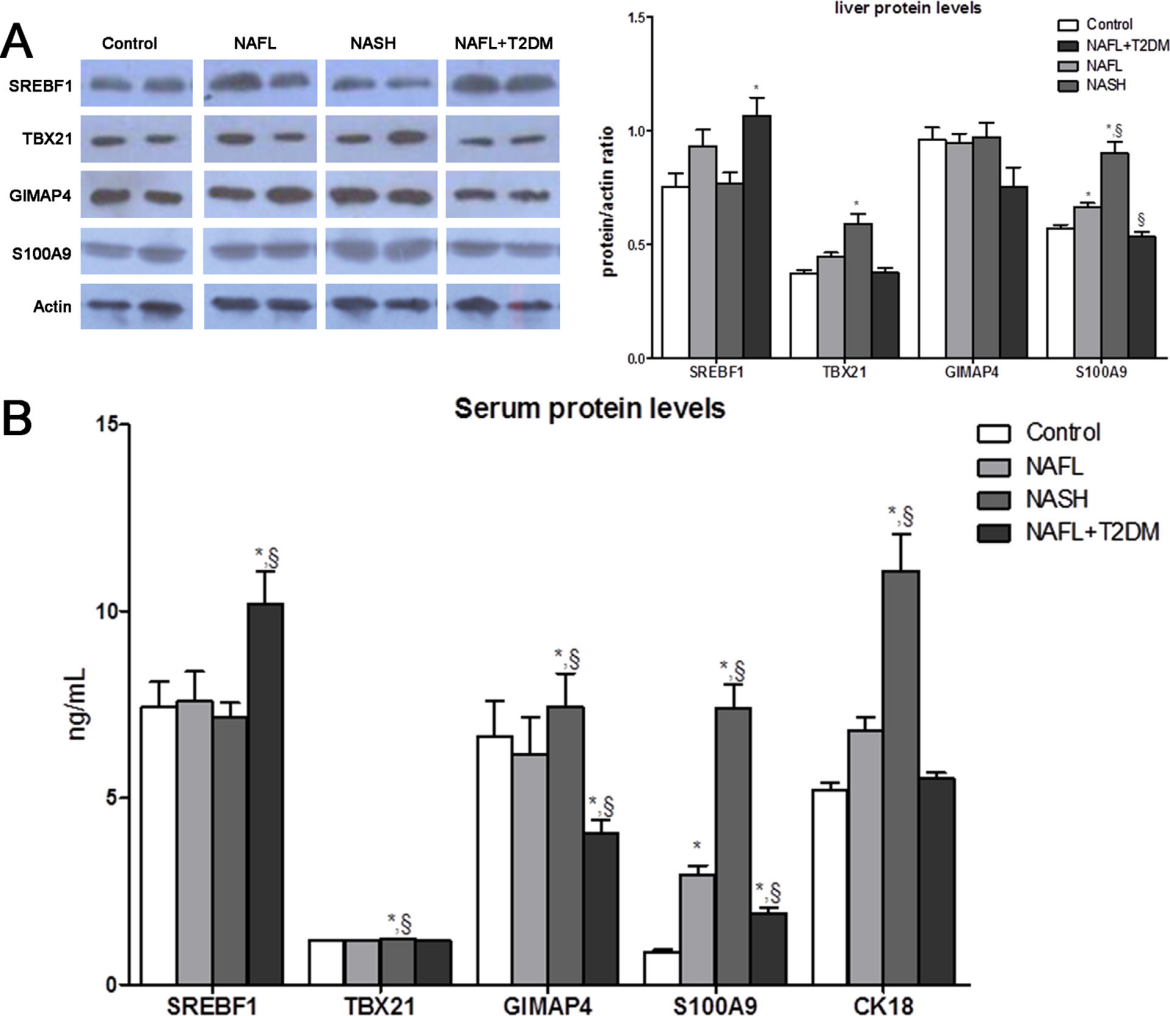
The levels of selected biomarkers in liver and serum. (A) Hepatic S100A9, SREBF1, TBX21, and GIMAP4 protein levels in control, NAFL, NASH and NAFL + T2DM groups. (B) Serum levels of SREBF1, TBX21, GIMAP4, S100A9, and CK18 protein in the control, NAFL, NASH, NAFL+T2DM groups. *, *P* < 0.05 compared with the control group; §, *P* < 0.05 compared with the NAFL group.

Since srebf1, tbx21, gimap4 and s100a9 was differentially regulated in the progression of NAFLD, we determined protein levels of these factors from both liver and serum in our rat models. Western blot results showed that hepatic levels of S100A9 were significantly increased in the NAFL and NASH groups compared with control (*P* < 0.05); however, S100A9 was slightly decreased in the NAFL + T2DM group compared with the NAFL group (*P* < 0.05). Meanwhile, SREBF1 showed an increase in NAFL + T2DM group, and TBX21 had an increase in NASH group when compared with control group (*P* < 0.05). But there was no significant difference in GIMAP4 among four groups (Fig 4A). Similarly, serum levels of SREBF1 and TBX21 were increased in NAFL + T2DM and NASH groups respectively compared with control and NAFL group (*P* < 0.05). There was an increase in NASH group and a decrease in NAFL + T2DM group of GIMAP4 serum level compared with control and NAFL group. We found that serum levels of S100A9 were increased in all 3 experimental groups, and a 2.5-fold increase was detected in the NAFL group compared with the control group. Additionally, there was a 4-fold increase in the NASH group and a 0.5-fold decrease in the NAFL + T2DM group compared with the NAFL group. As it is well documented that CK18 is one of potential markers of NASH, we measured its serum level in our rat models. Results showed that CK18 increased significantly in NASH group, but there was no significant difference in NAFL and NAFL + T2DM group compared with control (Fig 4B). Overall, only serum levels of S100A9 were increased in all the experiment groups, and compared with the NAFL group, levels in the NASH group were elevated, and those in the NAFL + T2DM group were decreased. This stable and consistent difference in hepatic and serum S100A9 expression among different phenotypes of NAFLD demonstrates its potential to be used as a biomarker to monitor NAFLD progression.

### Correlation between S100A9 and hepatic histologic features as well as biochemical indexes and its role in the non-invasive diagnosis of NASH

**Fig 5:**
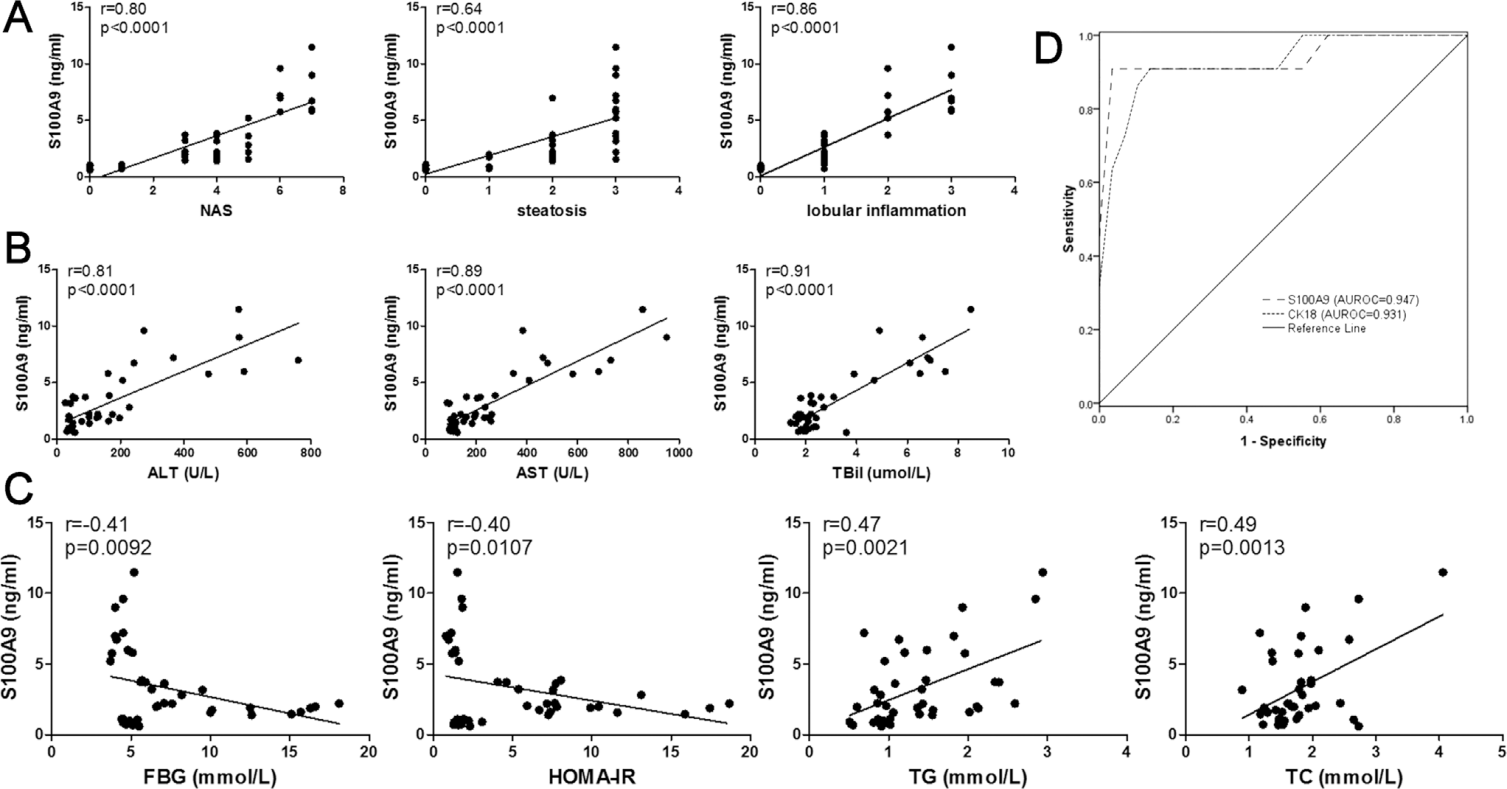
Linear regression analysis between serum S100A9 level and some clinical parameters, and its diagnostic value in definite NASH. Correlation between serum S100A9 level and (A) NAS, steatosis, lobular inflammation, (B) ALT, AST, TBil, (C) FBG, HOMA-IR, TG and TC in rats of all groups. (D) The receiver operating characteristic (ROC) curve of S100A9 and CK18 for the definite NASH diagnosis in rats. Area under the ROC (AUROC) for S100A9 and CK18 is 0.947 and 0.931 respectively.

To verify the practical value of S100A9 in clinical practice, we performed linear regression analysis between serum S100A9 levels and several histologic and biochemical parameters. We found that serum levels of S100A9 positively correlated with NAS and histologic features of hepatic steatosis and lobular inflammation (r = 0.80, 0.64, and 0.84, respectively, *P* < 0.001; Fig 5A), indicating that it is a reliable marker for the severity of hepatic histologic features. Biochemical indexes of liver injury, such as ALT, AST, and TBil also strongly correlated with serum S100A9 levels (r = 0.81, 0.89, and 0.91, respectively, *P* < 0.001; Fig 5B). FBG, HOMA-IR, TG, and TC, indicators of metabolic disorders, showed a weak correlation with serum S100A9 (r = -0.41, -0.40, 0.47, and 0.49, respectively, *P* < 0.05; Fig 5C). Data from this analysis show that S100A9 is more sensitive in NASH than in T2DM, which strongly suggests that S100A9 may be an ideal biomarker for hepatic progression in NAFLD but not for metabolic disorders.

We next tested the clinical value of serum S100A9 to diagnose definite NASH (NAS ≥ 5). The receiver operating characteristics (ROC) curve included rats within all groups and was established for various S100A9 and CK18 levels to evaluate the power of S100A9 to accurately predict NASH (Fig 5D). Interestingly, the area under the ROC (AUROC) curve of S100A9 was highly significant (0.947; 95% confidence interval, CI: 0.845–1.049). The AUROC of CK18 was 0.931 (95%CI: 0.836–1.026), which was even a little lower than that of S100A9. These findings unequivocally establish that the serum level of S100A9 is an excellent qualitative marker for the non-invasive diagnosis of NASH.

## Discussion

NAFLD is a complex disease with multiple sub-phenotypes and an intricate relationship with extra-hepatic complications [20]. Here, we provide evidence for distinct hepatic gene transcriptional signatures associated with different phenotypes of NAFLD, including the earliest stage (NAFL), advanced liver injury stage (NASH), and concomitant T2DM developed stage (NAFL+T2DM). We also provide the first set of data in the identification of advanced NAFLD with regard to hepatic and metabolic profiles. Finally, we demonstrate a role for S100A9 in the progression of NAFLD and its potential clinical value for non-invasive diagnosis of NASH.

NAFLD is a non-infectious, chronic, systemic, inflammatory pathological state. This is particularly true for NASH, which represents the most inflamed NAFLD condition, characterized by activation of inflammatory cells and up regulation of inflammatory mediators [21]. Previous work has established that S100A9 is an activator of toll-like receptor 4 (TLR4) and advanced glycation end products (AGER), and, as such, can stimulate innate immune cells and activate NF-kappa-B signaling pathway [22–24]. This effectively amplifies signaling via the proinflammatory cascade. In addition, PPAR and P53 have also been reported to regulate S100A9 [25, 26], and these factors are well known to play critical roles in the pathogenesis of NALFD. Although there is no data describing a role for S100A9 in NAFLD to date, it is reasonable to hypothesize that S100A9 may have a strong link with NAFLD. S100A9 is a secretory protein that is primarily released by cells of myeloid lineage, including neutrophils, monocytes, and dendritic cells [22]. Thus, we measured its levels in both rat liver and serum. The concordance of changes between S100A9 protein levels in liver and serum and its hepatic mRNA levels suggests that it may be useful as a reliable biomarker for the development of NAFLD, and it may possess the ability to differentiate different phenotypes of NAFLD.

Recently, there have been several publications exploring the relationship among obesity, atherosclerosis, and serum levels of S100A9. High circulating levels of S100A9 have been identified in patients with cardiovascular disease and diabetes [25, 27]. Here, we provide strong supporting evidence for high serum levels of S100A9 in different phenotypes of NAFLD. Although the NAFL + T2DM rat models have higher serum levels of S100A9 compared with controls, they showed decreased S100A9 levels compared with NAFL, suggesting that S100A9 may be more sensitive in NAFLD than in T2DM. It is also possible that some compensatory mechanisms are activated when concomitant T2DM develops in NAFL, and that NAFLD with hepatic progression may induce more secretion of S100A9 than T2DM. Furthermore, we find that S100A9 is effective in predicting hepatic pathologic features, particularly steatosis and lobular inflammation. It is also strongly correlated with several clinical parameters of liver injury, including ALT, AST, and TBil. Biochemical indexes of metabolic disorders, such as FBG, HOMA-IR, TG, and TC are weakly associated with serum S100A9 levels. This finding demonstrates that S100A9 is a more sensitive biomarker of hepatic progression in NAFLD compared with metabolic progression.

Several noninvasive, diagnostic biomarkers have been extensively examined for clinical benefit in NASH. Some of these are markers of inflammation, including ferritin, adipokines, and other cytokines, which have been shown to correlate with the presence and severity of NAFLD [28, 29]. Cytokeratin-18 (CK-18), a marker of apoptosis, also has potential value in differentiating NAFL from NASH and has a high specificity for NAFLD and fibrosis [30, 31]. The procollagen III N-terminal propeptide (PIIINP) is a marker of fibrosis and can discriminate between NAFL and NASH or advanced fibrosis [32]. CK-18 and PIIINP are better at distinguishing NASH from NAFL than inflammatory factors, but CK-18 exhibits limited sensitivity (sensitivity, 60%; specificity, 97.4%); PIIINP has poor specificity (sensitivity, 80%; specificity, 68%) [31, 32]. And our results showed that though CK18 had good quality in distinguishing NASH from control and NAFL, its value in differentiating NAFL from control was limited. Compared with these biomarkers, S100A9 not only has a higher AUROC in the diagnosis of NASH, but also shows a strong correlation with biochemical indexes and can distinguish different phenotypes of NAFLD.

In addition to S100A9, we identified three other factors, srebf1, tbx21, and gimap4, which can distinguish NASH and NAFL + T2DM. However, they were less sensitive and unable to distinguish NAFL from control. Srebf1 encodes a transcription factor named sterol regulatory element binding protein (SREBP), a key regulator involved in lipid metabolism and cholesterol biosynthesis; it has been shown to be a susceptibility gene in metabolic diseases, such as T2DM [33]. Although significant evidence shows raised hepatic SREBP levels in NAFLD [34], we found a significant decrease in SREBP levels in the NASH group in our experiments. A lack of insulin resistance in our MCDD-induced NASH model may explain these results [15]. Tbx21 is a transcription factor that induces differentiation of Th1 cells and the expression of IFN-γ [35]; gimap4 is up-regulated by Th1-inducing cytokines [36]. These two genes have been reported to play a role in immune-related diseases, such as autoimmune hepatitis [15], hepatitis B [37], and some extra-hepatic diseases. However, to the best of our knowledge, there is no data linking these genes to NAFLD. The work we present here is the first to demonstrate significant up-regulation of tbx21 and gimap4 in NASH, representing a condition of chronic inflammation.

There are some limitations of our study that should be noted. First, the identification of the practical value of S100A9 in NAFLD is just the first step, and further studies are required to illustrate the specific function of S100A9 in NALFD and its corresponding molecular mechanisms. Additionally, we only obtained information from rat models of different NAFLD stages, which may not fully reflect the features of NAFLD progression. Thus, human studies are needed to assess the practical value of S100A9 in natural history of NAFLD, and to examine whether S100A9 is a disease-specific biomarker in NAFLD.

In conclusion, our experiments have demonstrated that serum levels of S100A9 can differentiate hepatic and metabolic progression in NAFLD, as well as distinguish NAFL from the control, which highlights its potential value as a biomarker in NAFLD progression. The most intriguing finding is that S100A9 shows a significant positive correlation with hepatic histologic features and biochemical indexes that are indicative of liver injury. Therefore, S100A9 also has enormous potential as a biomarker for the non-invasive diagnosis of NASH.

## Supporting Information

S1 Fig The heatmap of significantly changed genes in different groups. A subset of differential genes was selected for clustering analysis. An intensity filter was used to select genes where the difference between the maximum and minimum intensity values exceeds 40000 among all microarrays. For this microarray project, the number of genes clustered was 229. The heatmap labels: A, control group; B, NASH group; C, NAFL group; D, NAFL+T2DM group.
(TIF)

S1 Table Score of hepatic pathological features in each rat model. The hepatic pathological features of steatosis (0–3), lobular inflammation (0–2) and hepatocellular ballooning (0–2) were scored according to the Nonalcoholic Steatohepatitis Clinical Research Network. NAS was calculated by adding the scores of steatosis, lobular inflammation and hepatocellular ballooning. NAS, NAFLD Activity Score.
(DOCX)
