# Supplementary figures and images for: S100A9: A Potential Biomarker for the Progression of Non-Alcoholic Fatty Liver Disease and the Diagnosis of Non-Alcoholic Steatohepatitis
Source: PLoS One. 2015 May 19;10(5):e0127352. doi: 10.1371/journal.pone.0127352 (PMC4437778; doi:10.1371/journal.pone.0127352)

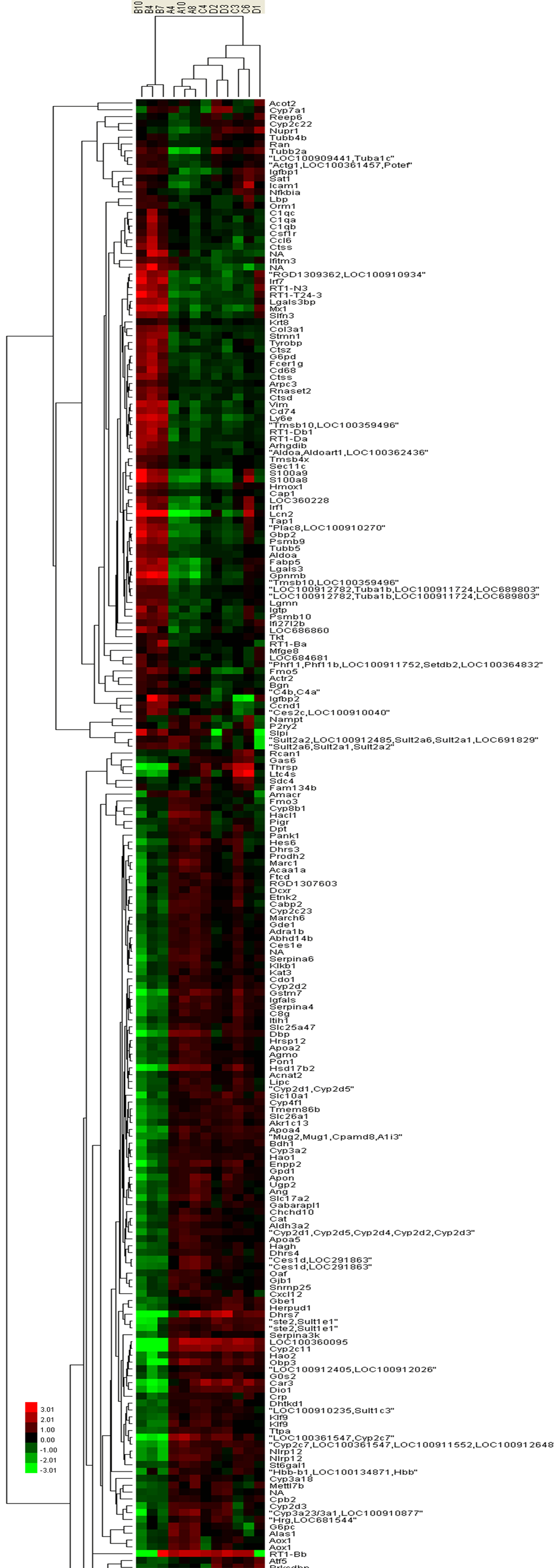

Supplement: S1 Fig — A subset of differential genes was selected for clustering analysis. An intensity filter was used to select genes where the difference between the maximum and minimum intensity values exceeds 40000 among all microarrays. For this microarray project, the number of genes clustered was 229. The heatmap labels: A, control group; B, NASH group; C, NAFL group; D, NAFL+T2DM group. (TIF) [file pone.0127352.s001.tif]
