# Supplementary material for: S100A9: A Potential Biomarker for the Progression of Non-Alcoholic Fatty Liver Disease and the Diagnosis of Non-Alcoholic Steatohepatitis
Source: PLoS One. 2015 May 19;10(5):e0127352. doi: 10.1371/journal.pone.0127352 (PMC4437778; doi:10.1371/journal.pone.0127352)
Supplement: S1 Table — The hepatic pathological features of steatosis (0–3), lobular inflammation (0–2) and hepatocellular ballooning (0–2) were scored according to the Nonalcoholic Steatohepatitis Clinical Research Network. NAS was calculated by adding the scores of steatosis, lobular inflammation and hepatocellular ballooning. NAS, NAFLD Activity Score. (DOCX) [file pone.0127352.s002.docx]

**S1 Table. Score of hepatic pathological features in each rat model**

| **Group** | **Steatosis** | **Lobular inflammation** | **Hepatocellular ballooning** | **NAS** |
| --- | --- | --- | --- | --- |
| **Control** | 1 | 0 | 0 | 1 |
|  | 0 | 1 | 0 | 1 |
|  | 0 | 1 | 0 | 1 |
|  | 0 | 0 | 0 | 0 |
|  | 0 | 0 | 0 | 0 |
|  | 0 | 0 | 0 | 0 |
|  | 0 | 0 | 0 | 0 |
|  | 1 | 0 | 0 | 1 |
|  | 0 | 1 | 0 | 1 |
|  | 0 | 0 | 0 | 0 |
| **NAFL** | 2 | 1 | 0 | 3 |
|  | 3 | 1 | 0 | 4 |
|  | 3 | 1 | 0 | 4 |
|  | 2 | 1 | 0 | 3 |
|  | 2 | 1 | 1 | 4 |
|  | 3 | 1 | 0 | 4 |
|  | 2 | 1 | 1 | 4 |
|  | 2 | 1 | 0 | 3 |
|  | 2 | 1 | 0 | 3 |
|  | 2 | 2 | 0 | 4 |
| **NASH** | 3 | 2 | 1 | 6 |
|  | 3 | 2 | 0 | 5 |
|  | 3 | 3 | 1 | 7 |
|  | 2 | 3 | 1 | 6 |
|  | 3 | 3 | 1 | 7 |
|  | 3 | 3 | 1 | 7 |
|  | 3 | 3 | 1 | 7 |
|  | 3 | 3 | 1 | 7 |
|  | 3 | 2 | 1 | 6 |
|  | 3 | 2 | 1 | 6 |
| **NAFL + T2DM** | 1 | 1 | 1 | 3 |
|  | 1 | 1 | 1 | 3 |
|  | 2 | 1 | 1 | 4 |
|  | 2 | 1 | 1 | 4 |
|  | 2 | 1 | 1 | 4 |
|  | 2 | 1 | 0 | 3 |
|  | 1 | 1 | 1 | 3 |
|  | 2 | 1 | 1 | 4 |
|  | 3 | 1 | 1 | 5 |
|  | 3 | 1 | 0 | 4 |

The hepatic pathological features of steatosis (0-3), lobular inflammation (0-2) and hepatocellular ballooning (0-2) were scored according to the Nonalcoholic Steatohepatitis Clinical Research Network. NAS was calculated by adding the scores of steatosis, lobular inflammation and hepatocellular ballooning.

NAS, NAFLD Activity Score.
